# Development of diabetic retinopathy after cataract surgery

**DOI:** 10.1371/journal.pone.0202347

**Published:** 2018-08-22

**Authors:** Chi-Juei Jeng, Yi-Ting Hsieh, Chung-May Yang, Chang-Hao Yang, Cheng-Li Lin, I-Jong Wang

**Affiliations:** 1 Department of Ophthalmology, Shuang-Ho Hospital-Taipei Medical University, New Taipei City, Taiwan; 2 Graduate Institute of Clinical Medicine, College of Medicine, National Taiwan University, Taipei, Taiwan; 3 Department of Ophthalmology, National Taiwan University Hospital, School of Medicine, Taipei, Taiwan; 4 Management Office for Health Data, China Medical University, Taichung, Taiwan; 5 Graduate Institute of Clinical Medical Science, China Medical University, Taichung, Taiwan; University of Colorado Denver School of Medicine, UNITED STATES

## Abstract

This study explored whether cataract surgery precipitates diabetic retinopathy (DR) development in diabetic patients without previous DR. Patients with the diagnosis of type II diabetes but without DR were selected from the Longitudinal Health Insurance Database 2000. Patients who received cataract surgery between January 1, 2000, and December 31, 2010, were included in the case group, and the control group was matched to the case group by age, sex, and index year. The postoperative incidence rates of nonproliferative diabetic retinopathy (NPDR), proliferative diabetic retinopathy (PDR), and diabetic macular edema (DME) were the main outcomes studied and were adjusted by age, sex, comorbidities, and statin, fibrate, angiotensin-converting-enzyme inhibitor (ACEI), oral hypoglycemic agents (OHA), and insulin use. In our cohort, patients who had dyslipidemia and used statins were more likely to undergo cataract surgery. Among diabetic patients without previous DR, patients receiving cataract surgery had a higher risk of NDPR development (adjusted hazard ratio = 1.48, 95% confidence interval = 1.15–1.91). No statistical difference was observed in PDR or DME development between operative and nonoperative groups. In additional stratified analyses, female sex, older age, comorbidities, surgery within 5 years, statin, ACEI, OHA, and insulin use increased the risk of NPDR development. In an adjusted Cox regression model, cataract surgery, OHA and insulin use were found to be risk factors for NPDR development. Cataract surgery with complications increased post-operative risks for NPDR were even higher, and the significant influence from cataract surgery persisted 5 years after surgery.

## Introduction

Between 1990 and 2010, diabetic retinopathy (DR) was ranked as the fifth most common cause of moderate-to-severe visual impairment worldwide [1]. Because its impact on working-age adults is profound, identifying risk factors for DR prevention and control is an important socioeconomic issue [2, 3]. Risk factors for DR include duration of diabetes, earlier age of onset of diabetes, presence of neuropathy, and elevated systolic blood pressure, cholesterol, and glycated hemoglobin A1C (HbA1C) [2, 4]. In addition to these systemic factors, cataract extraction has been also identified as an important ocular factor associated with DR progression [5, 6].

Cataracts may develop at an earlier age and may have a higher prevalence rate in patients with diabetes due to hyperglycemia [7–9] and the compromised blood–aqueous or blood–retina barriers [10, 11]. Breakdown of these barriers may also worsen postoperative inflammation after cataract surgery in both extracapsular cataract extraction and phacoemulsification, and this vicious cycle may instigate or expedite DR progression [12, 13]. Consequently, many studies have debated the relationship between cataract formation and DR progression and their risk factors. Henricsson et al identified higher HbA1C, duration of diabetes, insulin treatment, and existence of macular edema as the risk factors for DR progression after cataract extraction [14]. Hong et al noticed that the rate of DR progression almost doubled after phacoemulsification 12 months postoperatively. However, less progression was reported for phacoemulsification than for intracapsular cataract extraction and extracapsular cataract extraction (ECCE) [15]. In a paired-eye study, Jaffe et al found that nonproliferative diabetic retinopathy (NPDR) progressed in 7 of 19 eyes following ECCE, whereas none progressed in the other eye without operation during the follow-up period of 18 months [6]. Regarding diabetic macular edema (DME) progression after cataract surgery, Dowler et al suggested that cataract surgery accelerates DR and DME progression [16]. By contrast, Early Treatment Diabetic Retinopathy Study (ETDRS) data showed no significant difference in the incidence of clinically significant macular edema (CSME) before and after surgery [17]. Krepler et al and Romero-Aroca et al found no significant difference in DME occurrence at 12 months following phacoemulsification in patients with mild-to-moderate NPDR[18, 19]. Similarly, Biró and Balla found no significant difference in macular thickness after phacoemulsification in normal individuals and patients with diabetes within 2 months after surgery [20]. In this study, however, patients with severe NPDR or proliferative diabetic retinopathy (PDR) were specifically excluded. Squirrell et al concluded that DR progression might merely be the natural course in patients with varying degrees of DR, including PDR; they found no association between cataract surgery and the increased incidence of CSME [21]. This controversy might be due to the smaller sample size or shorter follow-up duration in these studies. This study investigated whether DR development or progression is affected by cataract surgery in a 10-year population-based cohort.

## Methods

### Data source

In 1995, the government of Taiwan launched the National Health Insurance (NHI) program, which covers more than 99% of the country’s population [22]. The National Health Research Institutes maintain the NHI Research Database (NHIRD) for research purposes. The Longitudinal Health Insurance Database 2000 (LHID2000) is one of the databases included in the NHIRD. The LHID2000 contains the data of one million patients randomly selected from the NHI program, including the patient’s sex, birth date, disease codes, and medical records. Each patient’s identification number is re-encoded to protect the patient’s privacy. This study was approved by the International Review Board (IRB) of China Medical University and Hospital (IRB permit number: CMUH-104-REC2-115).

### Sampled participants

The study cohort comprised patients with type II diabetes (International Classification of Diseases, Ninth Revision, Clinical Modification [ICD-9-CM] codes 250.x0 and 250.x2) and without DR (ICD-9-CM code 362.02). Among these patients, those who had received cataract surgery between January 1, 2000, and December 31, 2010, were included in the case group, and those who had never received cataract surgery were included in the control group. The index date was the date on which the patients received cataract surgery. The control group was then frequency matched to the case group patients by age, sex, and index year.

### Outcome, relevant variables, and comorbidities

Three events were considered in this study. The first event was NPDR (ICD-9-CM codes 249.5, 250.5, 362.01, 362.03–06, 362.07, 362.1, 362.81, and 362.82). The second event was DME (ICD-9-CM codes 362.53, 362.83, and 362.07), which was confirmed through the presence of codes for intravitreal injection treatment. The last event was PDR *(*ICD-9-CM codes 362.02 and 379.23), which was confirmed through the presence of codes for panretinal photocoagulation treatment. The diagnosis of NPDR, PDR, and DME was made at subsequent two visits with the same diagnosis. The diagnosis of DME or the administration of IVI treatment rely on the results of OCT (optical computer tomography) or FAG (fluorescein angiography) requested by the Taiwan National Health Insurance Program in insurance claimants on a reimbursement.

Hypertension (ICD-9-CM codes 401−405), dyslipidemia (ICD-9-CM code 272, A code A182), diabetic nephropathy (ICD-9-CM codes 249.4 and 250.4), diabetic neuropathy (ICD-9-CM codes 357.2, 249.60, and 249.61), heart disease (ICD-9-CM codes 410–429, A code A270, A279–A281, and A289), cardiovascular disease (ICD-9-CM codes 430–438), and peripheral arteriolar disease (ICD-9-CM codes 440–448, except for 440.1) were considered comorbidities in this study. Whether each patient had been prescribed statins, fibrates, or angiotensin-converting-enzyme inhibitors (ACEIs) was also considered.

We defined patients having either new diagnosis of retained lens material, cystoid mcular edema, or endophthalmitis (ICD9-CM codes 998.82, 998.82, 998.89, 997.99, 360.00–03, 362.52), or surgical intervention including pars plana vitrectomy, anterior vitrectomy, or intraocular lens reposition or exchange within three months after phacoemulsification or ECCE as having complicated cataract surgery.

### Statistical analysis

Table 1 shows the demographics of the two groups and a comparison of the differences between them. Differences were analyzed using a chi-squared test for categorical variables and a Student’s *t* test for continuous variables. The incidence rate of each event was calculated in person-years. Univariable and multivariable Cox proportional hazard regression models were used to estimate the hazard ratio (HR) and 95% confidence interval (CI). The variables included in the multivariable Cox model were age, sex, comorbidities, and the medications listed in Table 1. A Kaplan–Meier curve showed the cumulative incidence of NPDR for each group, and the differences between the two groups were analyzed using the log-rank test. Data analysis was performed using SAS statistical software (Version 9.4 for Windows; SAS Institute, Inc., Cary, NC, USA). Statistical significance was defined as a *P* value less than 0.05.

**Table 1:** Comparison of demographics and comorbidity between patients who received cataract surgery and those who did not (among diabetic patients without diabetic retinopathy).

	Cataract surgery		
	No (N = 1912)	Yes (N = 1912)	
	N (%)	N (%)	p-value
Age, years			0.99
≤64	498 (26.1)	498(26.1)	
≥65	1414 (74.0)	1414 (74.0)	
Mean (SD) ^†^	69.7 (9.86)	70.2 (9.68)	0.10
Gender			0.99
Female	1104 (57.7)	1104 (57.7)	
Male	808 (42.3)	808 (42.3)	
Comorbidity			
Hypertension	1433 (75.0)	1446 (75.6)	0.63
Dyslipidemia	1095 (57.3)	1219 (63.8)	<0.001
Diabetic nephropathy	122 (6.38)	130 (6.80)	0.60
Diabetic neuropathy	34 (1.78)	40 (2.09)	0.48
Heart disease	945 (49.4)	1006 (52.5)	0.05
Cardiovascular disease	236 (12.3)	198 (10.4)	0.05
Peripheral arteriolar disease	152 (7.95)	170 (8.89)	0.29
Medication			
Statin	552 (28.9)	672 (35.2)	<0.001
Fibrate	410 (21.4)	451 (23.6)	0.11
ACEI	999 (52.3)	1030 (53.9)	0.32
OHA	929 (48.6)	903 (47.2)	0.40
Insulin	282 (14.8)	254 (13.3)	0.19

The chi-squared test was used to examine categorical data.

^†^The *t* test was used to examine continuous data.

ACEI = angiotensin converting enzyme inhibitor; OHA = oral hypoglycemic agents.

## Results

A total of 1916 patients were included in the case group, and 1916 patients were included in the control group (Table 1). Using a chi-squared test, we found no differences in the distributions of sex and age between the two groups. Moreover, using the chi-squared test, no significant difference was observed in age distribution. Similarly, the distributions of the comorbidities of hypertension, diabetic nephropathy, diabetic neuropathy, and peripheral arteriolar disease were not significantly different. Regarding other comorbidities, more patients in the case group had dyslipidemia and heart disease, whereas more patients in the control group had cardiovascular disease. Regarding medication use, more patients in the case group were statin users. Only dyslipidemia and statin use were statistically significant after Bonferroni correction.

Table 2 shows the HR of NPDR, PDR, and DME between the case and control groups. Among diabetic patients without DR, the patients receiving cataract surgery had a higher risk of NPDR development (adjusted HR = 1.48, 95% CI = 1.15–1.91); however, no statistical differences were observed in PDR and DME between the patients who received cataract surgery and those who did not. We also stratified the patients by sex, age, comorbidity, medications, and follow-up period to compare the risk of NPDR development between the patients who received cataract surgery and those who did not. The patients with any one of the comorbidities in Table 1 were classified as the comorbidity group. We found that among the patients who received cataract surgery, women (adjusted HR = 1.68, 95% CI = 1.18–2.38), those aged ≥65 years (adjusted HR = 1.54, 95% CI = 1.13–2.09), the comorbidity group (adjusted HR = 1.48, 95% CI = 1.12–1.89), statin users (adjusted HR = 2.02, 95% CI = 1.20–3.42), ACEI (angiotensin converting enzyme inhibitor) users (adjusted HR = 1.57, 95% CI = 1.12–2.20), OHA (oral hypoglycemic agents) users (adjusted HR = 1.48, 95%CI-1.12–1.97), non-insulin users (adjusted HR = 1.74, 95% CI-1.28–2.36) had an increased risk for NPDR development. Patients operated ≤ 1 year have increased risks developing NPDR (adjusted HR = 2.58, 95% CI = 1.55–4.31), and the effect is significant upto 5 years (adjusted HR = 1.77, 95% CI = 1.32–2.37). Either using fibrate has increased risk of developing NPDR (nonuser adjusted HR = 1.35, 95% CI = 1.01–1.81; user adjusted HR = 2.08, 95% CI = 1.20–3.62).

**Table 2:** Incidence and adjusted hazard ratio of nonproliferative diabetic retinopathy, proliferative diabetic retinopathy, and diabetic macular edema by sex, age, and comorbidity between patients who received cataract surgery and those who did not (among diabetic patients without diabetic retinopathy).

	Cataract surgery						Compared to Control	

	No			Yes				
Variables	Events n	PY	Rate^#^	Events n	PY	Rate^#^	Crude HR (95% CI)	Adjusted HR^†^ (95% CI)
NPDR								
All	100	9010	11.1	147	9204	16.0	1.44 (1.12, 1.86)**	1.48 (1.15, 1.91)**
Gender								
Female	50	5432	9.21	83	5538	15.0	1.63 (1.15, 2.32)**	1.68 (1.18, 2.38)**
Male	50	3579	14.0	64	3666	17.5	1.25 (0.87, 1.82)	1.26 (0.87, 1.83)
P for interaction								0.31
Age, years								
≤64	31	2614	11.9	46	2522	18.2	1.54 (0.98, 2.44)	1.28 (0.80, 2.04)
≥65	69	6397	10.8	101	6682	15.1	1.41 (1.04, 1.91)*	1.54 (1.13, 2.09)**
P for interaction								0.15
Comorbidity^§^								
No	7	1118	6.26	6	873	6.87	1.09 (0.37, 3.25)	1.20 (0.40, 3.67)
Yes	93	7892	11.8	141	8331	16.9	1.44 (1.11, 1.87)**	1.48 (1.14, 1.92)**
P for interaction								0.64
Medication								
Statin								
No	80	6757	11.8	98	6437	15.2	1.29 (0.96, 1.73)	1.34 (0.99, 1.80)
Yes	20	2254	8.87	49	2767	17.7	2.01 (1.19, 3.38)**	2.02 (1.20, 3.42)**
P for interaction								0.15
Fibrate								
No	82	7238	11.3	105	7234	14.5	1.29 (0.96, 1.72)	1.35 (1.01, 1.81)*
Yes	18	1772	10.2	42	1969	21.3	2.11 (1.21, 3.66)**	2.08 (1.20, 3.62)**
P for interaction								0.12
ACEI								
No	45	4626	9.73	55	4478	12.3	1.26 (0.85, 1.87)	1.30 (0.88, 1.93)
Yes	55	4384	12.6	92	4726	19.5	1.56 (1.12, 2.18)**	1.57 (1.12, 2.20)**
P for interaction								0.42
Anti-DM drug								
No	19	5051	3.76	29	5202	5.57	1.49 (0.83, 2.65)	1.61 (0.90, 2.88)
Yes	81	3959	20.5	118	4002	29.5	1.44 (1.09, 1.92)*	1.48 (1.12, 1.97)**
P for interaction								0.93
Insulin								
No	66	7988	8.26	112	8211	13.6	1.65 (1.22, 2.24)**	1.74 (1.28, 2.36)***
Yes	34	1022	33.3	35	992	35.3	1.06 (0.66, 1.70)	1.02 (0.63, 1.66)
P for interaction								0.13
Follow-up period								
≦1 >1 ≦5	21 79 71	1857 7153 6821	11.3 11.0 10.4	50 97 124	1867 7337 6950	26.8 13.2 17.8	2.37 (1.42, 3.94)*** 1.20 (0.89, 1.61) 1.72 (1.28, 2.30)***	2.58 (1.55, 4.31)*** 1.20 (0.89, 1.62) 1.77 (1.32, 2.37)***
>5	29	2189	13.3	23	2254	10.2	0.77 (0.45, 1.34)	0.79 (0.46, 1.37)
PDR								
All	1	9357	0.11	2	9779	0.20	1.97 (0.18, 21.7)	1.68 (0.15, 18.8)
DME								
All	2	9350	0.21	8	9770	0.82	3.88 (0.82, 18.3)	3.83 (0.81, 18.1)

PY, person-years; Rate^#^, incidence rate, per 1,000 person-years; Crude HR: relative hazard ratio

Adjusted HR^†^: adjusted hazard ratio controlled for age; sex; and comorbidities of hypertension, dyslipidemia, diabetic nephropathy, diabetic neuropathy, heart disease, cardiovascular disease, and peripheral arteriolar disease; and statin, fibrate, ACEI drug use, OHA, and insulin

Comorbidity^§^: Patients with any one of the comorbidities of hypertension, dyslipidemia, diabetic nephropathy, diabetic neuropathy, heart disease, cardiovascular disease, and peripheral arteriolar disease were classified as part of the comorbidity group

*P < 0.05

**P < 0.01

***P < 0.001

We further discussed the risk factors for NPDR for all diabetic patients without DR in Table 3. Using an adjusted Cox regression model, cataract surgery, taking OHA or using insulin were determined to be risk factors for NPDR. Patient with diabetic nephropathy and neuropathy did not show increased risk after adjustment with OHA and insulin. Patients using OHA and insulin have higher risks developing NDPR (in OHA group, adjusted HR = 4.54, 95% CI = 3.23–6.38; in insulin group, adjusted HR = 1.45, 95% CI = 1.07–1.96).

**Table 3:** Hazard ratios of nonproliferative diabetic retinopathy in association with sex, age, and comorbidities in univariable and multivariable Cox regression models.

	NPDR	
Variable	Crude HR (95% CI)	Adjusted HR^†^ (95% CI)
Cataract surgery	1.44 (1.12, 1.86)**	1.48 (1.15, 1.91)**
Gender (Women vs Men)	1.29 (1.01, 1.65)*	1.24 (0.96, 1.60)
Age, years	0.99 (0.98, 1.01)	0.99 (0.97, 1.00)
Baseline comorbidities (yes vs no)		
Hypertension	1.36 (1.003, 1.85)*	1.09 (0.75, 1.58)
Dyslipidemia	0.96 (0.75, 1.24)	0.93 (0.69, 1.25)
Diabetic nephropathy	2.53 (1.74, 3.67)***	1.36 (0.92, 2.01)
Diabetic neuropathy	2.86 (1.64, 5.01)***	1.59 (0.90, 2.81)
Heart disease	0.97 (0.75, 1.24)	0.98 (0.75, 1.29)
Cardiovascular disease	1.45 (1.00, 2.11)	1.21 (0.82, 1.78)
Peripheral arteriolar disease	0.80 (0.47, 1.34)	0.82 (0.48, 1.39)
Medications		
Statin	0.99(0.75, 1.31)	0.79 (0.58, 1.08)
Fibrate	1.22 (0.91, 1.63)	1.01 (0.73, 1.39)
ACEI	1.45 (1.12, 1.87)**	1.05 (0.77, 1.44)
OHA	5.28 (3.86, 7.24)***	4.54 (3.23, 6.38)***
Insulin	3.05 (2.31, 4.03)***	1.45 (1.07, 1.96)*

Crude HR: relative hazard ratio; Adjusted HR†: adjusted hazard ratio controlled for age; sex; and comorbidities of hypertension, dyslipidemia, diabetic nephropathy, diabetic neuropathy, heart disease, cardiovascular disease, and peripheral arteriolar disease; and statin, fibrate, ACEI use, OHA, and insulin

*P < 0.05

**P < 0.01

***P < 0.001

**Fig 1:**
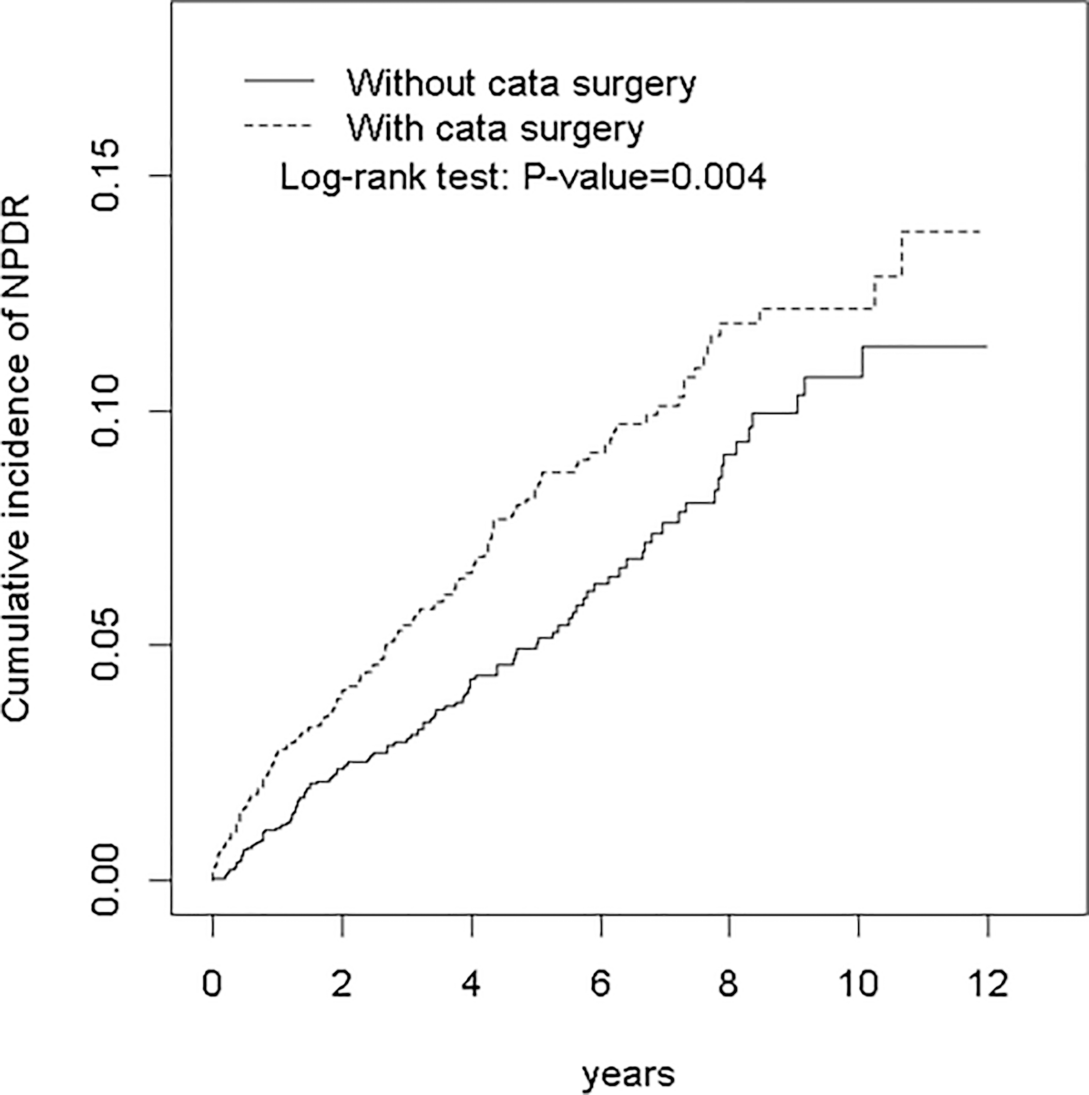
Cummulative incidence of NPDR between patients who received cataract surgery and those who did not (among diabetic patients without diabetic retinopathy).

As illustrated in Fig 1, the Kaplan–Meier curve showed that the cumulative incidence of NPDR was higher in the case group than in the control group (log-rank test, *P* = 0.004).

Table 4 showed cataract surgery with complications are at even higher risk developing NPDR (with complications, adjusted HR = 5.40; 95% CI = 2.70–10.8; without complications, adjusted HR = 1.42, 95% CI-1.10–1.84).

**Table 4:** Incidence, and hazard ratio of NPDR between patients who received cataract surgery with complications within three months and those who did not (among diabetic patients without diabetic retinopathy).

	Event	PY	Rate^#^	Crude HR (95% CI)	Adjusted HR† (95% CI)
None	100	9010	11.1	1 (Reference)	1 (Reference)
Cataract surgery					
Without complications	138	9017	15.3	1.38 (1.07, 1.79)*	1.42 (1.10, 1.84)**
With complications	9	187	48.1	4.28 (2.17, 8.47)***	5.40 (2.70, 10.8)***

PY, person-years; Rate#, incidence rate, per 1,000 person-years; Crude HR: relative hazard ratio

Adjusted HR†: adjusted hazard ratio controlled for age; sex; and comorbidities of hypertension, dyslipidemia, diabetic nephropathy, diabetic neuropathy, heart disease, cardiovascular disease, and peripheral arteriolar disease; and statin, fibrate, ACEI drug use, OHA, and insulin

*P < 0.05

**P < 0.01

***P < 0.001

## Discussion

Regarding the risk factors for cataract formation in patients with diabetes, the rate of cataract formation has been reported to be influenced by age [7, 23, 24], severity of preoperative DR [7, 14, 25, 26], duration of diabetes [7, 14], HbA1c level, and other factors [7, 14, 27]. Klein et al reported that among patients with diabetes, women had a higher rate of cataract extraction than men [7]. However, in our study, we found no significant differences in age distribution, hypertension, diabetic nephropathy, diabetic neuropathy, and peripheral arteriolar disease between the two groups. By contrast, more patients in the case group had dyslipidemia and used statins. Therefore, we believe that both dyslipidemia and statin use are strongly associated with cataract formation requiring surgery. Although the relationship between statin use and cataract formation in patients with and without diabetes [28] is still under debate, our study finding implies that dyslipidemia and statin use may accelerate cataract formation. Our results are distinct from those of previous studies in that our cohort was followed up for 10 years and comprised patients with good compliance for medications and treatments according to the records available. Therefore, other risk factors for cataracts requiring surgery may have been well adjusted in our cohort.

Based on the results of many studies, dyslipidemia is still controversial as a risk factor for cataract formation. Donnelly et al found that total cholesterol is lower in patients with cataract [29]. In an Israeli cohort, diabetes and hyperlipidemia were found to be independently related to a higher incidence of cataract formation [30]; the results were consistent with those of a later Korean study [31]. In the Singapore Malay Eye study, researchers found low high-density lipoprotein to be associated with cortical cataracts [32]. These results indicate dyslipidemia and diabetes should be considered as risk factors for cataract formation both independently and dependently. Similarly, the Beaver Dam Eye Study showed that statin use was negatively associated with nuclear cataract formation [33]. The Blue Mountains Eye Study (BMES) showed that statin use reduced the risk of nuclear or cortical cataract formation [34]. By contrast, our results confirmed the role of dyslipidemia in cataract formation among diabetic patients. Differences between our study and the Beaver Dam Eye Study and the BMES may be attributed to differences in ethnicities and the incidence of dyslipidemia in patients with diabetes. Therefore, the Taiwanese population should more closely mirror the populations in the Korean study and the Singapore Malay Eye Study.

Ostri et al reported that postoperative corrected visual acuity in patients with diabetes receiving cataract surgery was affected by the degree of DR and age, particularly in patients with a history of focal laser treatment for CSME [35]. In the ETDRS, the operated eyes had a higher trend toward two-step DR progression than the other eye, but this difference was not statistically significant. Moreover, the proportion of eyes developing CSME was not markedly different whether or not lens extraction was performed [17]. A prospective study found that DR development and progression was not influenced by lens surgery, but DR development and progression followed its natural course [36]. By contrast, in the paired-eye study by Chung et al, the operated eye showed greater progression than the nonoperated eye and was affected by poor renal function and preoperative CSME [37]. Hong et al also reported an increase in the doubling rate of DR progression 12 months after surgery [15]. Hausser et al noted that male sex and the duration of diabetes were associated with DR occurrence, and that sugar control was related to DR progression after phacoemulsification [38]. Notably, a meta-analysis of different paired-eye studies showed that the progression rate of DR and the incidence rate of DME increased significantly after phacoemulsification.[39] Another meta-analysis showed that phacoemulsification influenced macular thickness in patients with mild-to-moderate DR, but not in patients without DR, and the difference persisted until 6 months postoperatively [40]. In our study, we found that cataract surgery increased the risk of NPDR development, and the risks are significant 1 year post-operatively, and its influence was significant upto 5 years. Regarding the risk factors, female sex, age more than 65 years, certain comorbidities, statin use, and ACEI use led to a higher risk of NPDR development in patients with diabetes who underwent cataract surgery. Also, patients receiving OHA, patients not using insulin for sugar control are ate higher risk developing NPDR after cataract surgery. In previous studies, cataract surgery may induce the elevation of vascular endothelial growth factor, monocyte chemotactic protein-1, interleukin-1β (IL-1β), and IL-6 in the aqueous fluid of patients with and without diabetes by causing changes to the blood–retinal barrier [41, 42]. Moreover, our results confirm those of previous studies, which have shown the incidence of DME or CSME did not increase after cataract surgery in diabetic patients without DR. According to adjusted Cox regression model, cataract surgery, using OHA, and using insulin were risk factors for NPDR in all patients with diabetes (Table 3). The effect of diabetic nephropathy and diabetic neuropathy on NPDR development were not significant after adjusting with OHA and insulin control. These results further confirm that cataract surgery is a risk factor affecting the progression from no DR to NPDR. Patients requiring OHA and even insulin for sugar control are associated with DR development. In a prospective study conducted by Henricsson et al., patients shifting from oral hypoglycemic agents to insulin have higher relative risk of progression. The risk factors for progression in their study was higher HbA1C and moderate NPDR at baseline. Those using insulin at baseline are not at risk otherwise[43]. In a meta-analysis, the association between DR and insulin was weakened after adjusting with DM duration[44]. Synergistic effect of insulin with vascular endothelial growth factor (VEGF) were proposed for higher risks of DR development in patients using insulin[45]. Insulin is also a growth hormone[46], where the effect from insulin on DR development was more obvious at puberty, young people, or pre-existing neovascularization. Sugar control cannot be retrieved directly from our database. Insulin and OHA are both significant risk factors for NDPR development. Whether the risk is from higher blood glucose requiring further insulin, or from insulin itself cannot be deduce from our study directly.

Surgical inexperience and longer surgical duration were associated with faster DR progression post-operatively in previous studies[47]. Cataract complications may produce stronger and prolonged inflammation post-operatively. Inflammation is one of the DR pathogenesis, which leads to breakdown of blood-retinal barriers and increases oxidative stress[48]. In our study, patients with complicated surgery or repeat surgery within 3 months have obviously higher risks developing NPDR than those without complications.

In conclusion, cataract surgery significantly increases the risk of NPDR development. Its impact persists 5 years after surgery. The risk developing NPDR post-operatively are even higher if there is complications during cataract surgeries. Diabetic patients receiving cataract surgery who have comorbidities are more susceptible to NPDR development. Whether cataract surgery accelerates DME or PDR development needs further investigation.
